# *Phytophthora agathidicida* was undetected in a pilot survey of soils outside the host's ( *Agathis australis* ) natural range

**DOI:** 10.17912/micropub.biology.002181

**Published:** 2026-07-02

**Authors:** Isabella CR Hadlow, Jade TT Palmer, Monica L Gerth

**Affiliations:** 1 School of Biological Sciences, Victoria University of Wellington, New Zealand

## Abstract

Surveillance of *Phytophthora agathidicida* , the causal agent of kauri dieback, has largely been restricted to kauri ( *Agathis australis* ) forests. To assess the pathogen’s wider environmental distribution in New Zealand, we applied a highly sensitive, species-specific endpoint PCR assay to soil from non-forested locations south of the host’s natural geographic range. *P. agathidicida* was not detected in any samples. Successful positive, negative, extraction, and inhibition controls supported these results. These pilot findings demonstrate the absence of detectable *P. agathidicida* at the tested sites, providing additional data regarding the pathogen’s geographic range in New Zealand.

**Figure 1. Geographic distribution of sampling sites and PCR-based screening for P. agathidicida:** **(A)** **Map showing the 13 soil collection sites** . All sites were located south of latitude 38° S (grey dashed line), representing the southern limit of the natural *Agathis australis* (kauri) range. **(B) PCR screening for *Phytophthora agathidicida*** . No amplification of the *P. agathidicida* PA-LTR region (Palmer et al., 2025) was detected in any environmental sample. The positive control (PA+; 3 pg *P. agathidicida* genomic DNA) produced the expected ~161 bp product. **(C)** **Inhibition controls.** Parallel PA-LTR reactions were spiked with 1 pg *P. agathidicida* genomic DNA for each sample. All reactions produced the ~161 bp product, indicating no significant PCR inhibition. **(D)** **Extraction controls.** Successful extraction of DNA from all soil samples was confirmed using 18S rDNA primers (18S #3 and #5RC; Machida et al., 2012), with reactions yielding the expected ~350 bp product. All panels included a positive control (PA+) and a no-template control (NTC). PCR products were resolved on 2% TAE agarose gels stained with GelGreen (panels B and D) or GelRed (panel C). 100 bp, 100 bp DNA Ladder (New England Biolabs); LMW, Low Molecular Weight DNA Ladder (New England Biolabs). Map data © MapTiler and © OpenStreetMap contributors.

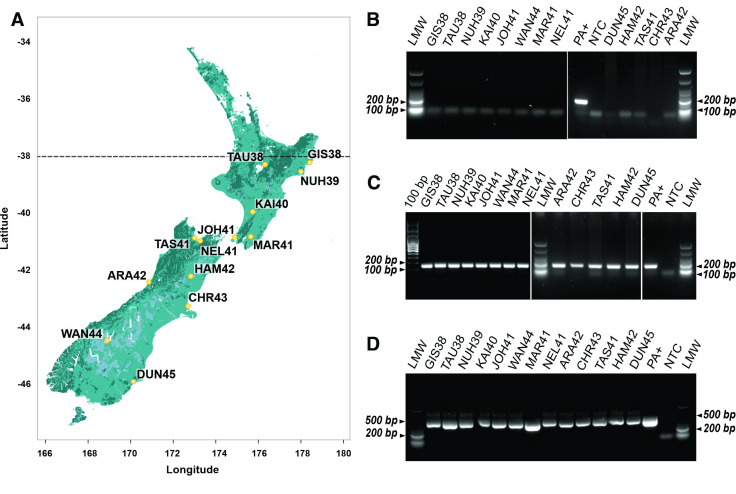



## Description

Numerous *Phytophthora* species are established in Aotearoa New Zealand, affecting forestry, agriculture and native ecosystems (Burgess et al., 2017; Cheah et al., 2008; Dick et al., 2014; Scott & Williams, 2014). Of major concern is *Phytophthora agathidicida,* which causes dieback disease in New Zealand’s northern kauri ( *Agathis australis* ) forests (Bradshaw et al., 2020; Mainwaring et al., 2023). Management of kauri dieback relies on an accurate understanding of the current distribution of *P.* *agathidicida* in soils, both within kauri forests and in surrounding landscapes.

To date, surveillance for *P. agathidicida* has been largely restricted to kauri forests and has primarily relied on soil baiting assays (Froud et al., 2021; Froud et al., 2025; Tiakina Kauri, 2023). However, soil baiting is resource-intensive (McDougal et al., 2014; Singh et al., 2017), which has limited the scale of monitoring efforts and precluded broader landscape-scale testing. Consequently, it remains unknown whether *P. agathidicida* persists in environments outside of kauri forests. While it is frequently presumed to be an introduced organism currently expanding its range, an alternative hypothesis suggests it may be a native species that is naturally widespread (Bradshaw et al., 2020; Hood, 2021). Determining its presence or absence in non-kauri landscapes is therefore critical for testing these biogeographical assumptions. The recent development of a species-specific, high-sensitivity PCR-based assay, capable of detecting as little as 1 fg of *P. agathidicida* DNA (Palmer et al., 2025), now makes larger-scale surveillance feasible. This diagnostic tool enables the investigation of the pathogen's broader distribution, facilitating testing in previously unexamined regions. Such data are vital for disease management, dispersal tracking, and long-term conservation planning.

In this pilot study, we investigated the presence of *P. agathidicida* in soils outside the natural latitudinal range of kauri (Steward & Beveridge, 2010). A panel of soil samples from 13 non-forested locations ( Figure 1A ) was processed using the double-filtration technique and screened for *P. agathidicida* via endpoint PCR (Palmer et al., 2025). None of the 13 samples tested positive for *P. agathidicida* ( Figure 1B ). The success of internal controls—including spiked *P. agathidicida* DNA to check for PCR inhibition ( Figure 1C ) and 18S rDNA amplification to confirm DNA extraction efficiency ( Figure 1D )—verified the reliability of these negative results.

Beyond the immediate results, the absence of amplification across these geographically diverse samples also provides practical evidence regarding the assay’s performance in complex soil matrices. Given that concerns regarding false-positive results are a primary barrier to the adoption of molecular tools for *P. agathidicida* , this study provides further evidence that the assay does not cross-react with common soil taxa in the environments sampled. This reinforces its potential as a tool for kauri dieback surveillance.

While we acknowledge the limited scope of this pilot study, the absence of detectable *P. agathidicida* DNA suggests that the pathogen was not present at the sampled sites. However, it is possible that low-abundance or spatially heterogeneous populations may fall below the assay’s detection limit of 1 fg and thus remain undetected. Furthermore, our sample size was small and restricted to human-modified, non-forested sites, with only a single sample collected per location. Future research should expand the ecological diversity of sampled sites, including indigenous forests where kauri are absent, to confirm the pathogen’s absence across a broader range of environmental conditions. Additionally, studies should incorporate multiple replicates per site to account for the potential spatial heterogeneity of *P. agathidicida* in soil. Nevertheless, these initial results provide a preliminary indication that *P. agathidicida* is not widely distributed beyond the natural range of its primary host.

## Methods

In this pilot study, soil samples were collected from private properties between June 2024 and July 2025. Participants were instructed to select a site that had not been disturbed by digging or soil amendment within the preceding six months, then remove loose debris and plant material from that site. Using a clean, inverted plastic bag, participants collected a large handful of soil from the top 5 cm of soil. Samples included fine roots when present. One sample per site was collected. The final sample set comprised soils from established yards, pastures, and plantings in both rural and suburban areas. These samples were sealed, double-bagged and transported at room temperature. Once received, the samples were mixed thoroughly by shaking and breaking up any soil aggregations without opening the sample bags. The homogenised samples were then aliquoted into 50 mL centrifuge tubes and stored at 4 °C until processing.

DNA was extracted from the samples using the double-filter bag method described by Palmer and Gerth (2025). Briefly, 5–10 g of soil was heat-sealed into a 90 μm nylon mesh pouch (120 x 60 mm), which was nested inside a 25 μm nylon mesh pouch (150 x 80 mm). Pouches were placed in 50 mL sterile water and subjected to wash cycles with vortexing at 2700 rpm to liberate oospores. Material >90 μm was discarded; the oospore-soil slurry captured by the 25 μm mesh was pelleted at 3000g for 2 min. The pellets were then resuspended in 800 μL Solution CD1 and transferred to Precellys 2 mL Soil Grinding Beads Kit (SK38) tubes. Following 10 min of homogenization using a Vortex-Genie 2 with a 24-tube adapter (Qiagen), DNA was extracted using the DNeasy PowerSoil Pro Kit (Qiagen) per the manufacturer's instructions. Eluted DNA was further purified via the DNeasy PowerClean Pro Cleanup Kit (Qiagen) to remove residual PCR inhibitors, and final elution was performed in 100 μL low-EDTA TE buffer (10 mM Tris-HCl, 0.1 mM EDTA) rather than the manufacturer’s supplied buffer to ensure greater DNA stability during storage.

Endpoint PCRs were performed in 40 μL reactions comprising 1× HOT FIREPol Blend Master Mix (Solis BioDyne), 0.5 µM of each primer, 25 µL PCR-grade water, and 5 µL of template DNA. PCR-grade water served as a negative control. Purified *Phytophthora agathidicida* genomic DNA (isolate NZFS 3770, 3 pg) served as a positive control. For the PA-LTR primer pair, the following PCR thermal cycling conditions were used: 95 °C for 12 min, followed by 40 cycles of 95 °C for 15 s, 63 °C for 40 s, and 72 °C for 7 s, and a final extension of 72 °C for 5 min. To monitor for PCR inhibition, parallel reactions were spiked with 1 µL of *P. agathidicida* genomic DNA at 1 pg/µL.

Extraction success was verified using eukaryotic 18S primers that have been used previously for broad-range taxonomic coverage in soil environmental DNA studies, including the detection of oomycetes (Drummond et al., 2015; Lear et al., 2018; Machida & Knowlton, 2012). For the 18S primer pair, the following PCR thermal cycling conditions were used: 95 °C for 12 min, followed by 30 cycles of 95 °C for 15 s, 63 °C for 30 s, and 72 °C for 60 s, and a final extension of 72 °C for 5 min.

For all replicates, controls performed as expected: negative controls produced no product; positive and inhibition controls produced the expected ~161 bp product; and extraction controls produced the expected ~350 bp product. The PCR products were visualised by electrophoresis on 2% agarose gels pre-stained with either GelRed or GelGreen (Biotium), run at 85 V for 35 minutes.

## Reagents

**Table:** 

**Primers**		
**Name**	**Sequence (5′ - 3′)**	**Reference**
18S #3	GYGGTGCATGGCCGTTSKTRGTT	Machida & Knowlton, 2012
18S #5RC	GTGTGYACAAAGGBCAGGGAC	Machida & Knowlton, 2012
PA-LTR-for	ACGCGCTCTGTTTCTTTAGC	Palmer et al., 2025
PA-LTR-rev	GCGGGCTTCCATTCAATTCA	Palmer et al., 2025

**Table:** 

**Kits and Reagents**	
**Name (Supplier)**	**Catalogue Number**
DNeasy PowerSoil Pro Kit (Qiagen)	47016
Soil grinding SK38 2 mL tubes (Bertin)	P000915-LYSK0-A
DNeasy PowerClean Pro Cleanup Kit (Qiagen)	12997-50
Qubit dsDNA High Sensitivity Assay Kit (Invitrogen)	Q32854
5× HOT FIREPol Blend Master Mix Ready to Load, 10 mM MgCl _2_ (Solis Biodyne)	04-25-00120-5
